# The potential value of cuprotosis in myocardial immune infiltration that occurs in pediatric congenital heart disease in response to surgery with cardiopulmonary bypass

**DOI:** 10.1002/iid3.795

**Published:** 2023-03-14

**Authors:** Song Puwei, Ma Siyu, DeQin ZhuoGa, Wu Kede, Yang Zhaocong, Nishant Patel, Liu Xiaoxu, Mo Xuming

**Affiliations:** ^1^ Department of Cardiothoracic Surgery Children's Hospital of Nanjing Medical University Nanjing China; ^2^ Department of Cardiothoracic Surgery, Nanjing Children's Hospital Medical School of Nanjing University Nanjing China

**Keywords:** CPB, cuproptosis, heart tissue injury, immune infiltration, inflammation

## Abstract

**Background:**

Cardiopulmonary bypass may cause malfunction in the myocardium. Cuproptosis is a novel cell death aggregating mitochondrial proteins. However, the research on cardiopulmonary bypass‐caused heart tissue injury in immune infiltration and cuproptosis is limited.

**Method:**

Immune infiltration, enrichment analysis, protein−protein interaction network, and medication prediction are applied to reanalysis differentially expressed genes and cuproptosis‐related genes in gene expression omnibus data set GSE132176.

**Results:**

Seven cuproptosis related genes (PDHA1, LIPT1, LIAS, DLST, DLD, DLAT, and DBT) and dendritic cells and Th1 cells are involved in heart tissue injury in response to surgery with cardiopulmonary bypass.

**Conclusions:**

Immune infiltration and cuproptosis are potential mechanisms by which cardiopulmonary bypass surgery may cause damage to heart tissue, which may be a new therapeutic target.

## Graphical abstract

Figure 8 shows the immune infiltration in the myocardial after cardiopulmonary bypass.

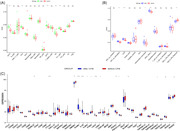

## 1 INTRODUCTION

The development of cardiopulmonary bypass (CPB) technology has enabled the treatment of complex congenital heart defects in neonates and newborns.^1^ During surgical procedures, the risk of dysfunction in the heart, the brain, and other vital organs increased^2^, ^3^ and the protection of the brain, lungs, kidneys, and heart following surgery with CPB has been a matter of intense interest.^4^, ^5^

Cuproptosis is a new kind of programmed cell death in which an accumulation of intracellular copper contributes to the aggregation of mitochondrial proteins and destabilization of Copper cluster proteins, most likely resulting in a specialized form of cell death.^6^ Previous studied shows that the myocardial copper concentrations increased during the aortic cross‐clamping period in the heart surgery.^7^ The change of myocardial copper ion concentration is closely related to the impact of cardiac surgery on myocardial cells. Therefore, we assume that copper death participates in and affects the process of myocardial injury during cardiopulmonary bypass. The study of copper toxicity and cuproptosis‐related genes (CRGs) is still in its infancy and there is an urgent need for more research in this field. Microarrays have been utilized in recent years to examine the expression of various organ differential genes (DEGs) before and after therapy with CPB. Liang and coworkers established a network regulatory relationship between Long noncoding RNA (LncRNA) and transcription factors in the injured rat brain after surgery with CPB^8^ and Ghorbel et al. concentrated on differential gene expression in the renal medulla using a well‐established model of acute kidney damage after surgery with CPB.^9^

We reanalyzed the data set based on Raggi et al.'s sequencing data to analyze tetralogy of Fallot (TOF) and atrial septal defect (ASD) specific myocardial transcriptional signatures in the gene expression omnibus (GEO) data set and bioinformatics permits us to explore how these genes function in the pathophysiology of heart tissue injury after CPB.^10^

## 2 METHODS AND MATERIALS

### 2.1 Data set collection and normalization

This microarray GSE132176 (https://www.ncbi.nlm.nih.gov/GEO/platform/GSE132176) is comprised of 10 right atrial samples with ASD and TOF for expression study before and after surgery with CPB and all samples were incleded in our study. Annotation of raw data is used to map probes to their associated gene symbols. When numerous probe sets are associated with a single gene, the expression levels of each probe within the multiple probe sets are averaged and ascribed to a single gene. The data were then Log2 transformed and *Z*‐score normalized. Networkanalysts were used to construct boxplots, principal component analysis (PCA) paragraph, count sum, density plot, and mean squared deviation (MSD) plots.^11^

### 2.2 Screening for DEGs and CRGs

DEGs were identified using the limma package in R. The ggplot2 and pheatmap packages were used to constructed volcano map and heatmap of DEGs, respectively. DEGs with *p* < .05 and |log_2_FC| >1 were considered as being significantly different.

The unpaired *T*‐test is used to examine the fragments per kilobase of transcript per million fragments mapped (FPKM) values of genes related with cuproptosis‐induced cell death.

### 2.3 Enrichment analysis and drug prediction of DEGs and CRGs

In our study, the “clusterProfiler” R package, metascape (metascape [http://metascape.org/gp/index.html]) and Enrichr (https://maayanlab.cloud) were used to perform Kyoto encyclopedia of genes and genomes (KEGG) pathway^12^ analysis, drug prediction from the DsigDB, and gene ontology (GO) analysis^13^ containing biological pathways (BP), cellular components (CC), and molecular function (MF).

Enrichr and metascape are web‐based tools for performing intuitive enrichment analysis with many visualization kinds and gene collection functionalities.^14^, ^15^

Gene set enrichment analysis (GSEA) version 4.1.0 software was used to analyze genes function from the GSEA website MsigDB database (http://software.broadinstitute.org/gsea/msigdb). The default weighted enrichment method was applied for enrichment analysis. The random combination was set for 1000 times. GO and KEGG pathway AND reactome pathway enrichment analysis were performed with GSEA and FDR < 0.25, NOM *p* < .05, and |NES| >1 were considered significant enrichment.

### 2.4 Protein−protein interaction (PPI) network construction and hub gene identification

Online database STRING (available online: http://string-db.org)^16^ was employed to develop DEGs‐encoded proteins and PPI network. Cytoscape software^17^ was used to establish a network of protein interaction relationships and to assess the protein interaction relationships of the putative DEGs. On Cytoscape, Molecular Complex Detection (MCODE)^18^ was used to identify functional clusters of genes in the PPI network with the parameters degree cutoff = 2, node score cutoff = 0.2, k‐core = 2, and maximum depth = 100. The proteins matching to the center nodes may be core proteins and candidate genes with crucial physiological regulatory roles.

### 2.5 Immune infiltration analysis and correlation analysis with cuproptosis

Using the “GSVA” and “ssGSEA” R packages, we can analyze the infiltration score of 16 distinct immune cells, the activity of 13 distinct immune‐related activities and the activity of 40 distinct immunological‐related checkpoints. In addition, correlation analyses between cuproptosis genes, immune cells, and immune‐related activities are generated and shown using the “corplot” and “heatmap” R packages.

### 2.6 Statistical analysis

As mentioned in the figure legends, the data are presented as means with standard deviations and the student's *t*‐test is used to examine significant differences. In addition, the Mann−Whitney test with *p* values adjusted by Benjamini−Hochberg is used to compare the ssGSEA scores of immune cells or pathways between the high risk and low risk groups. Moreover, the statistical significance level is set at *p* < .05. GraphPad Prism (Version 8.0.2), ggpubr, and reshape2 are also utilized to analyze the data and provide the appropriate graphics.

## 3 RESULTS

### 3.1 Standardization of data and identification of DEGs

The primary goal of normalization is to remove technical and systematic data variability so that comparisons may be made across samples.

**Figure 1:**
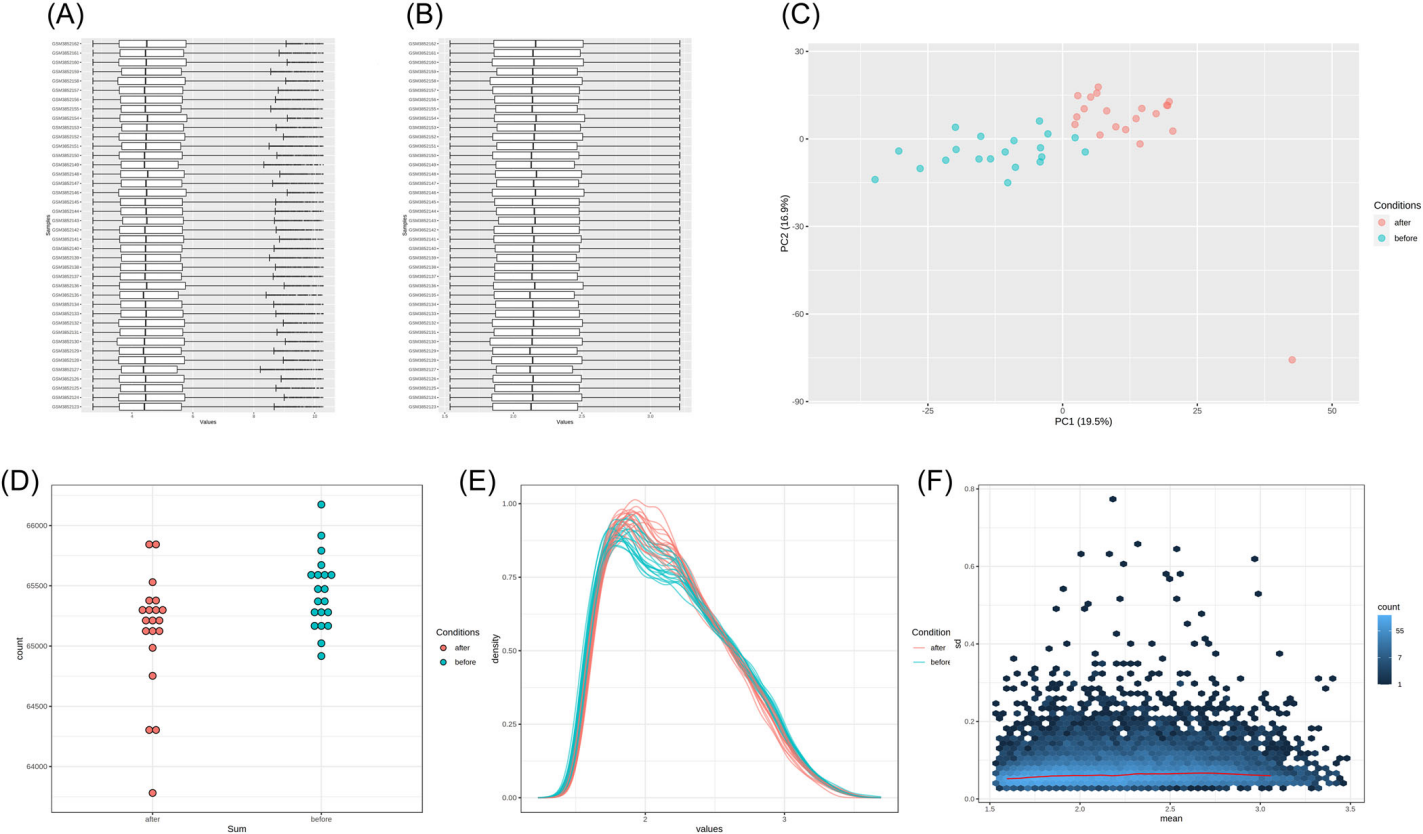
Normalization of data set GSE132176: (A & B). The boxplots of data distribution before and after normalization from GSE132176. (C) Principal component analysis of the combined data set after batch effect correction. (D) Probe counts were summed for heart spicies before and after CPB respectively. (E) Density plot facilites comparison of signals between arrays and identification of arrays with deviating distributions. (F) MSD plots were created for individual trajectories and fit to a straight line. CPB, cardiopulmonary bypass; MSD, mean squared deviation.

Probe counts (Figure 1D) were first summed for heart tissues before and after CPB respectively. Afterwords during normalizing the microarray data, the gene expressions of each sample are shown in the boxplot (Figure 1A,B) and the black lines in the boxes are virtually on the same straight line, verifying the raw data's correctness.

PCA (Figure 1C) paragraph which assess the biological variation across samples after normalization shows two group are distinguished apparently. In addition, the lines of two group in density plot (Figure 1E) which facilites comparison of signals between arrays and identification of arrays with deviating distributions depits similar to each other. MSD plots (Figure 1F) which created for individual trajectories fit it to a straight line which indicated further the potiential value of analysis.

**Figure 2:**
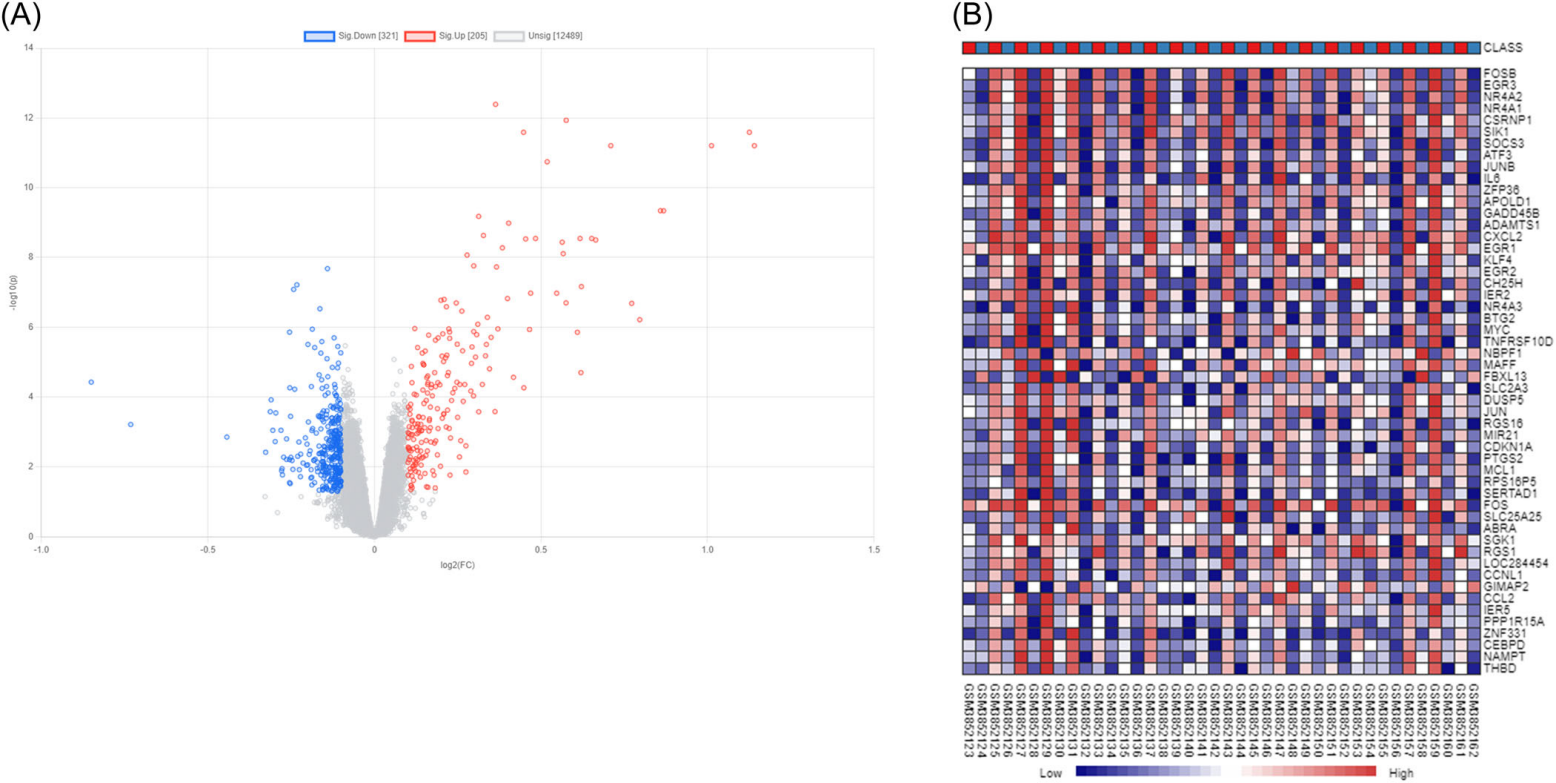
Screening of differential genes. (A) The volcano map shows the distribution of DEGs. Based on the GSE132176 database, 2937 DEGs were screened out, including 1504 upregulated DEGs and 1433 downregulated DEGs. Red stands for upregulated genes; blue stands for downregulated genes. (B) Heatmap of the top 50 up‐ and downregulated differential genes identified. DEGs, differential genes.

Significant DEGs were identified among post‐CPB heart tissues compared with before‐CPB ones. A total of 2937 DEGs were screened out, including 1504 upregulated DEGs and 1433 downregulated DEGs. These data were used to build the volcano plot of DEGs (Figure 2A) and the heatmap of the top 50 genes (Figure 2B).

### 3.2 Enrichment and PPI network analysis of the DEGs

**Figure 3:**
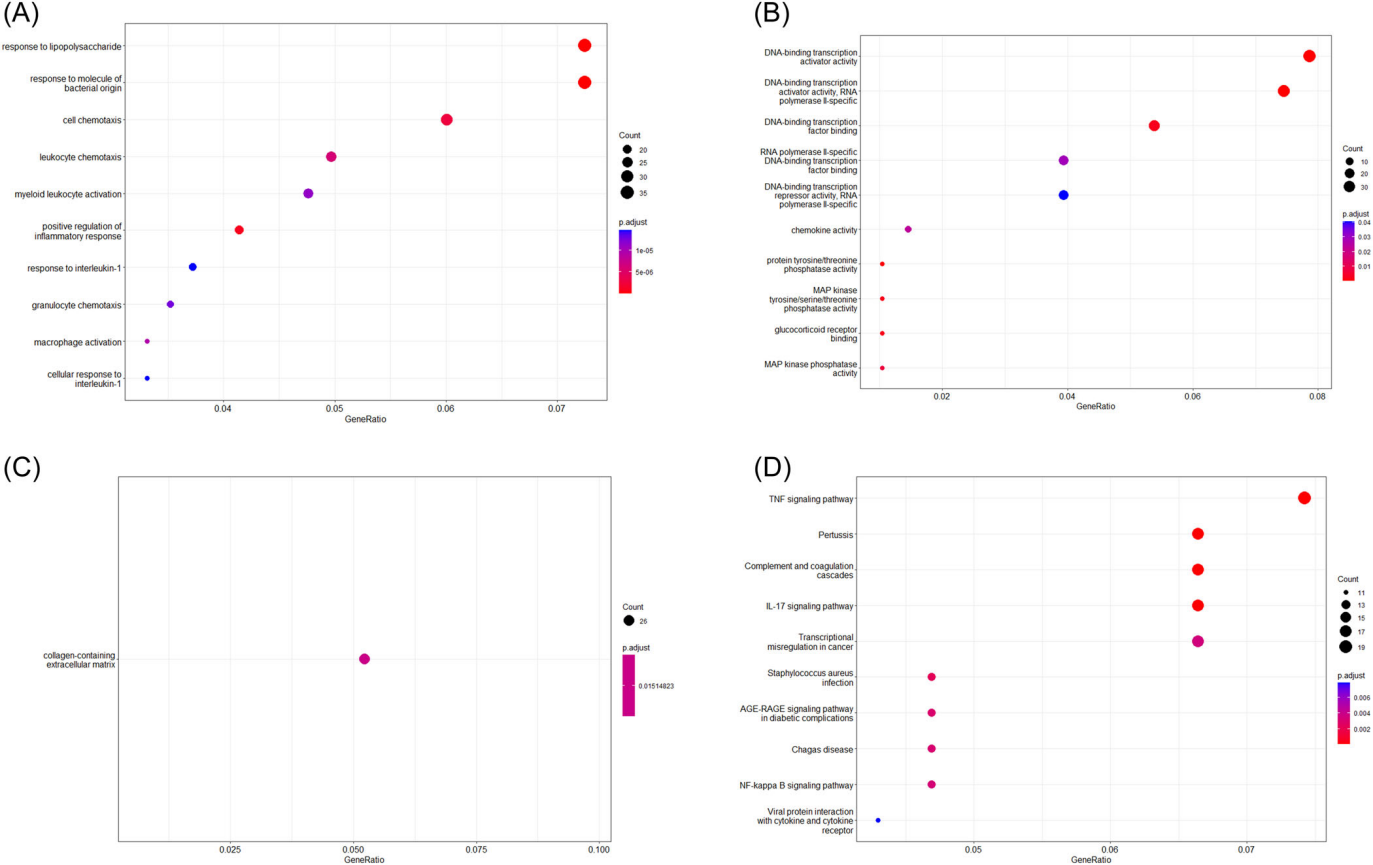
Pathway enrichment analysis of DEGs. (A−C) The enriched item in the gene ontology analysis of DEGs. (A) BP: biological process, (B) CC: cellular component, (C) MF: molecular function. (D) The enriched item in the Kyoto encyclopedia of genes and genomes analysis of DEGs. DEGs, differential genes.

The enrichment analysis revealed that there were markedly enriched in response to lipopolysaccharide (*p* < .01), response to molecule of bacterial origin (*p* < .01), cell chemotaxis (*p* < .01), and leukocyte chemotaxis (*p* < .01) in BP (Figure 3A) and there were also significant enriched in DNA‐binding transcription activator activity (*p* < .01), RNA polymerase II‐specific (*p* < .01), and DNA‐binding transcription factor activity (*p* < .01) in MF (Figure 3B). In the meanwhile, there were markedly enriched in collagen‐containing extracellular matrix (*p* < .01) in CC enrichment (Figure 3C). What's more there were markedly enriched in TNF signaling pathway (*p* < .01), pertussis (*p* < .01), and complement and coagulation cascades (*p* < .01) in KEGG pathway (Figure 3D).

**Figure 4:**
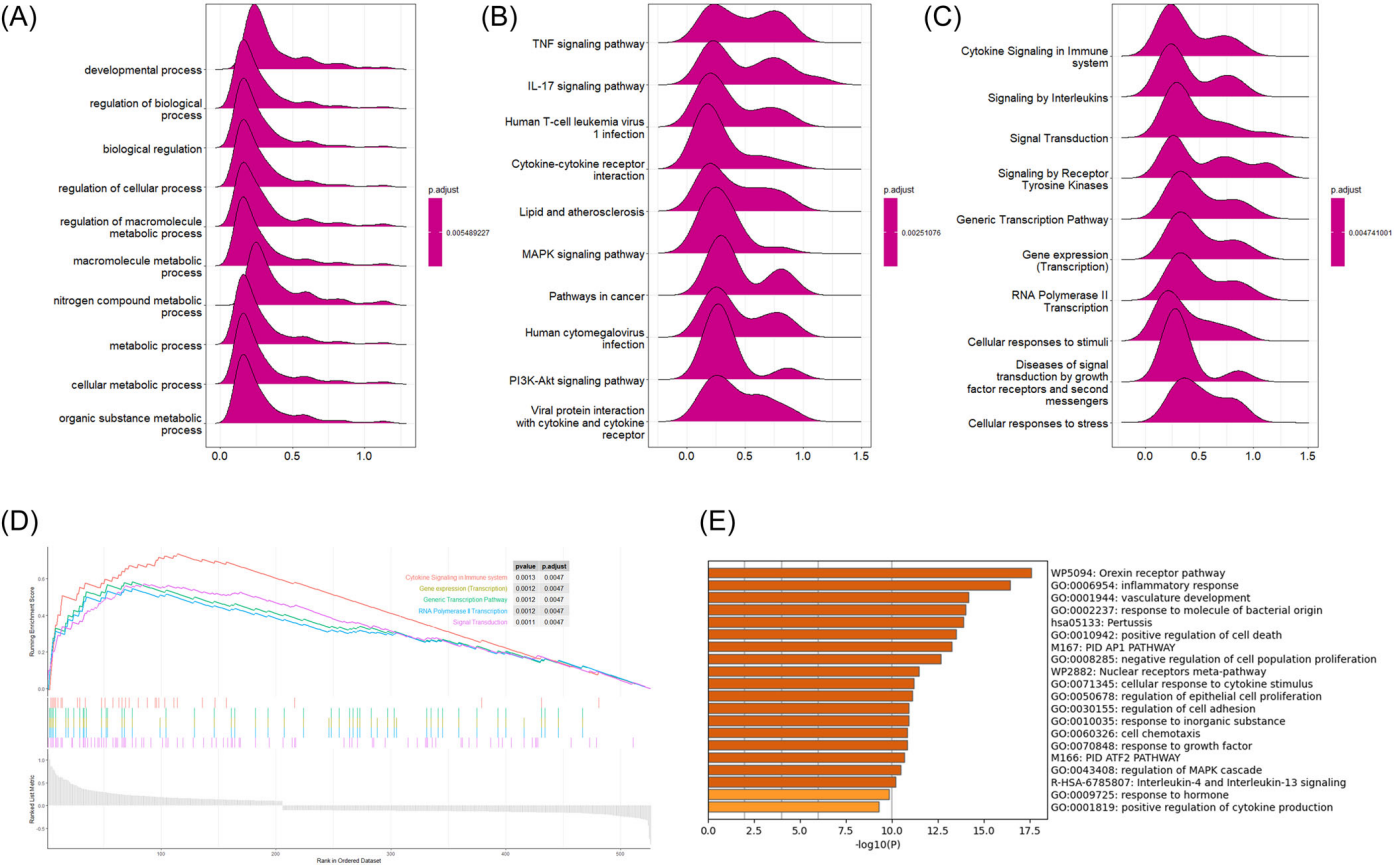
Gene set enrichment analysis (GSEA) and metascape enrichment analysis. (A−C) GSEA and metascape was used to analyze the signaling pathways enrichment in different groups. GSEA used to validate the gene signatures including GO (A) and KEGG (B) pathway and reactome pathway (C). (D) Metascape used to perform enrichment analysis. GO, gene ontology; KEGG, Kyoto encyclopedia of genes and genomes.

GSEA and metascape were also used to analyze the signaling pathways enrichment in different groups. There were markedly enriched in developmental process (*p* < .05, NES = 0.47, NOM *P* < .01, FDR < 0.01), regulation of biological process (*p* < .05, NES = 0.49, NOM *P* < .01, FDR < 0.01), and biological regulation (*p* < .05, NES = 0.47, NOM *P* < .01, FDR < 0.01) in GO enrichment (Figure 4A). Meanwhile, there were markedly enriched in TNF signaling pathway (*p* < .05, NES = 0.78, NOM *P* < .01, FDR < 0.01), IL‐17 signaling pathway (*p* < .05, NES = 0.74, NOM *P* < .01, FDR < 0.01), and human T‐cell leukemia virus 1 infection (*p* < .05, NES = 0.67, NOM *P* < .01, FDR < 0.01) in KEGG pathway (Figure 4B). What's more, there were markedly enriched in cytokine signaling in immine system (*p* < .05, NES = 0.7, NOM *P* < .01, FDR < 0.01), signaling by interleukins and signal transduction in GO reactome pathway (Figure 4C,D). There were markedly enriched in orexin receptor pathway (*p* < .01), inflammatory response (*p* < .01), and vasculature development in METASCAPE enrichment (Figure 4E).

**Figure 5:**
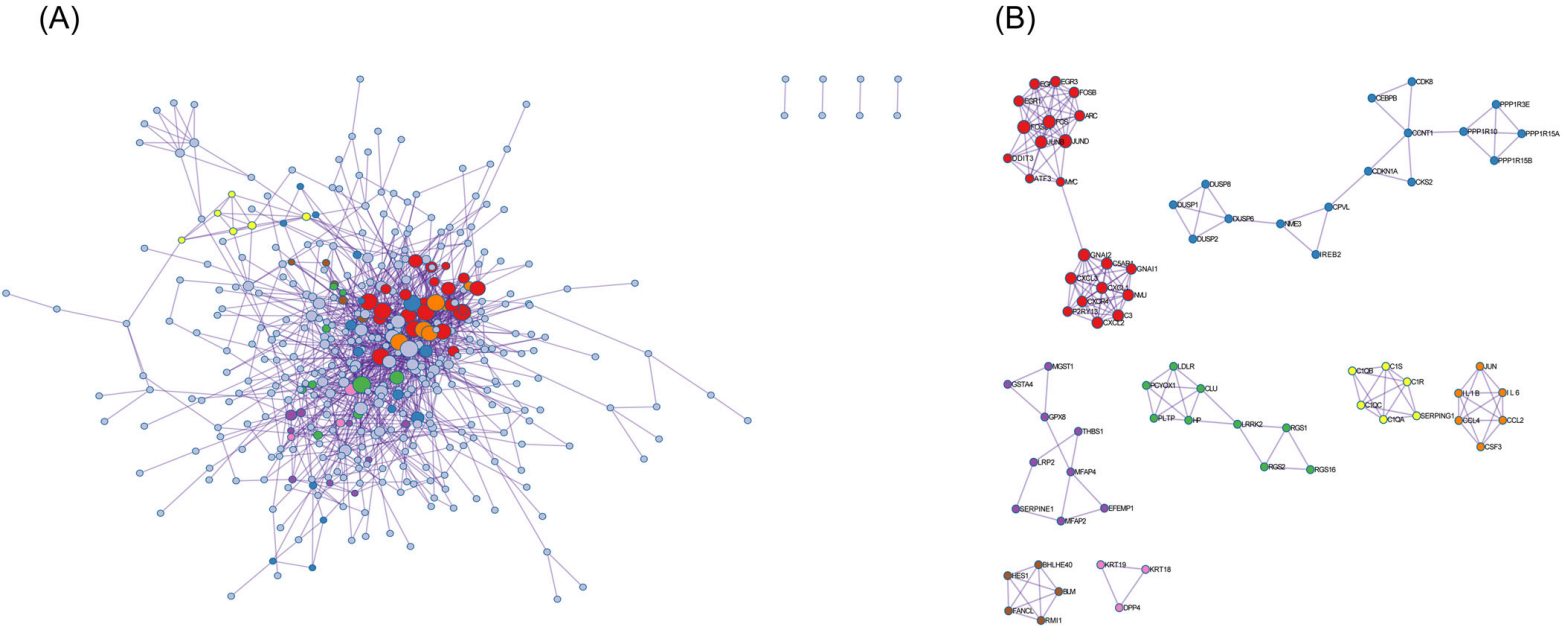
PPI network construction among DEGs and hub genes modules. (A) PPI network construction among DEGs. (B) The hub genes modules calculated by MCODE. DEGs, differential genes; MCODE, molecular complex detection; PPI, protein−protein interaction.

Afterwords, A PPI network of DEGs that included 406 nodes and 1097 interactions was constructed to identify gene interactions (Figure 5A). Module analysis identified significant clustering modules in the PPI network (Figure 5B). The three highest‐scoring clustering modules were obtained and each hub gene was found in ≥1 module. Thus, the eight clustering modules may represent key biological roles of the PPI network.

### 3.3 Identification and enrichment analysis of CRGs

**Figure 6:**
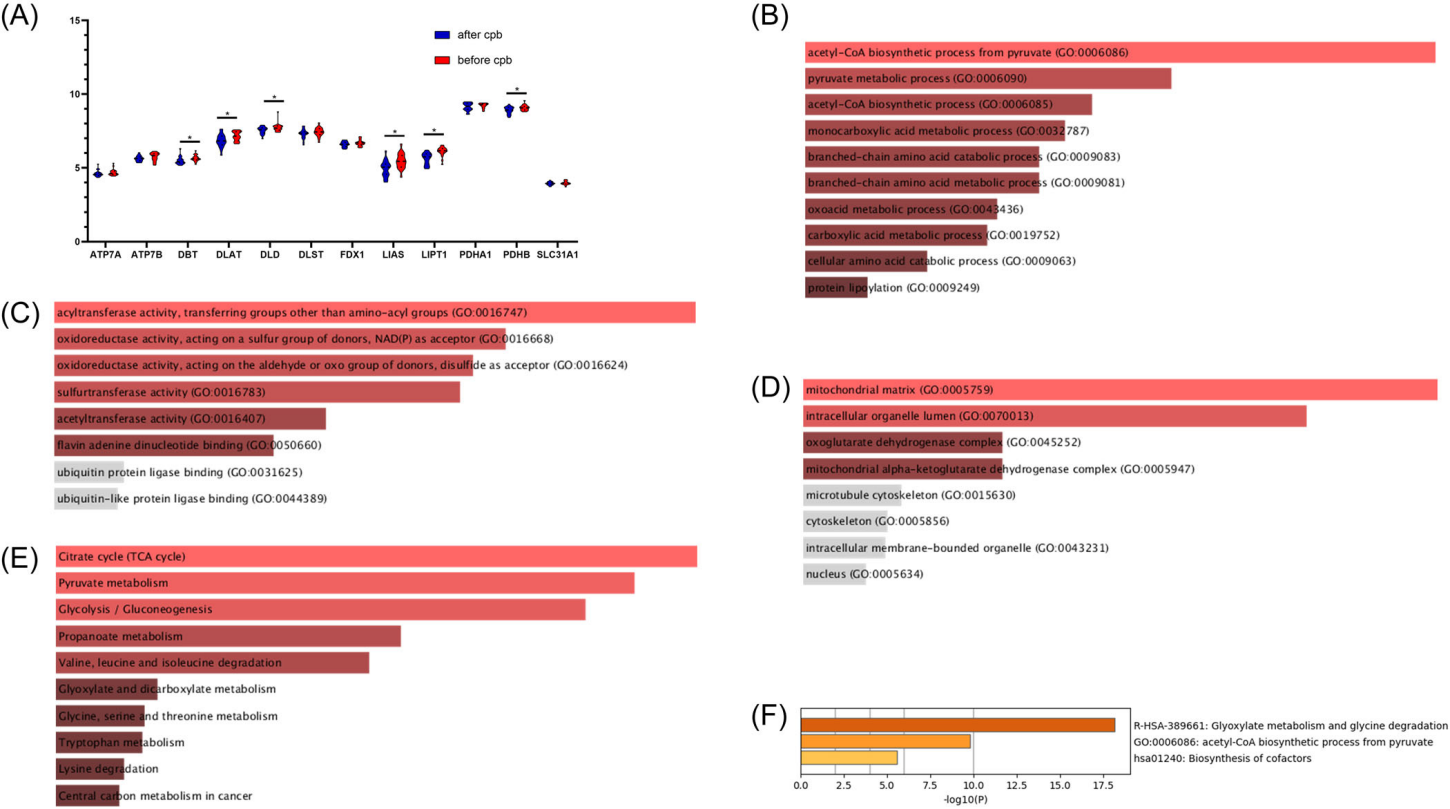
Identification and pathway enrichment analysis of DEGs. (A) Identification of cuproptosis according to fpkm value. Expression of DBT, DLAT, DLD, LIAS, LIPT, and PDHB in the myocardium of patients who had DHCA surgical therapy is lower than preoperatively. (B−D) The enriched item in the gene ontology analysis of DEGs. (B) BP: biological process, (C) CC: cellular component, (D) MF: molecular function. (E) The enriched item in the Kyoto encyclopedia of genes and genomes analysis of DEGs. (F) Metascape used to perform enrichment analysis. DEGs, differential genes.

According to previous study, ATP7A, ATP7B, DBT, DLAT, DLD, DLST, FDX1, LIAS, LIPT1, PDHA1, PDHB, and SLC31A1 are all substantially associated with cuproptosis^6^ and after idenfications it is shown that DBT (*p* < .05), DLAT (*p* < .05), DLD (*p* < .05), LIAS (*p* < .05), LIPT (*p* < .05), and PDHB (*p* < .05) gene expressions in the myocardium are lower after CPB operating procedure (Figure 6A).

Enrichr tool is used to perform enrichment analysis and predict the drugs with genes involved in copper‐related cell deaths and immune infiltration.

First, biochemical pathway primarily enriched in acetyl‐CoA biosynthesis from pyruvate (GO: 0006086) (*p* < .05), pyruvate metabolism (GO: 0006090) (*p* < .05), and cellular amino acid catabolic activity (GO: 0009063) (*p* < .05) (Figure 6B).

The majority of the enriched results in MF are for the following functions: acyltransferase activity, transferring‐groups other than amino—acyl groups (GO: 0016747) (*p* < .05), oxidoreductase activity, acting on a DONOR group, NAD (P) as acceptor (GO: 0016668) (*p* < .05), and oxidoreductase activity, acting on the aldehyde or oxo group of donors, disulfide as acceptor (GO: 0016624) (*p* < .05) (Figure 6C).

Also genes implicated in copper‐related cell death showed an enrichment in the CC categories as mitochondrial matrix (GO: 0005759) (*p* < .05), intracellular organelle lumen (GO: 0070013) (*p* < .05), and oxoglutarate dehydrogenase complex (GO: 0045252) (*p* < .05) (Figure 6D).

In the meanwhile, when analyzing genes involved in copper‐related cell death, the KEGG pathway enrichment analysis found that the citrate cycle (TCA cycle) (*p* < .05), pyruvate metabolism, and glycolysis/gluconeogenesis (*p* < .05) are most heavily involved (Figure 6E).

The metascape enrichs in glyoxylate metabolism (*p* < .05) and glycine degradation, acetyl‐CoA biosynthesis from pyruvate, and biosynthesis of cofactors (*p* < .05) (Figure 6F).

### 3.4 Immune infiltration analysis

**Figure 7:**
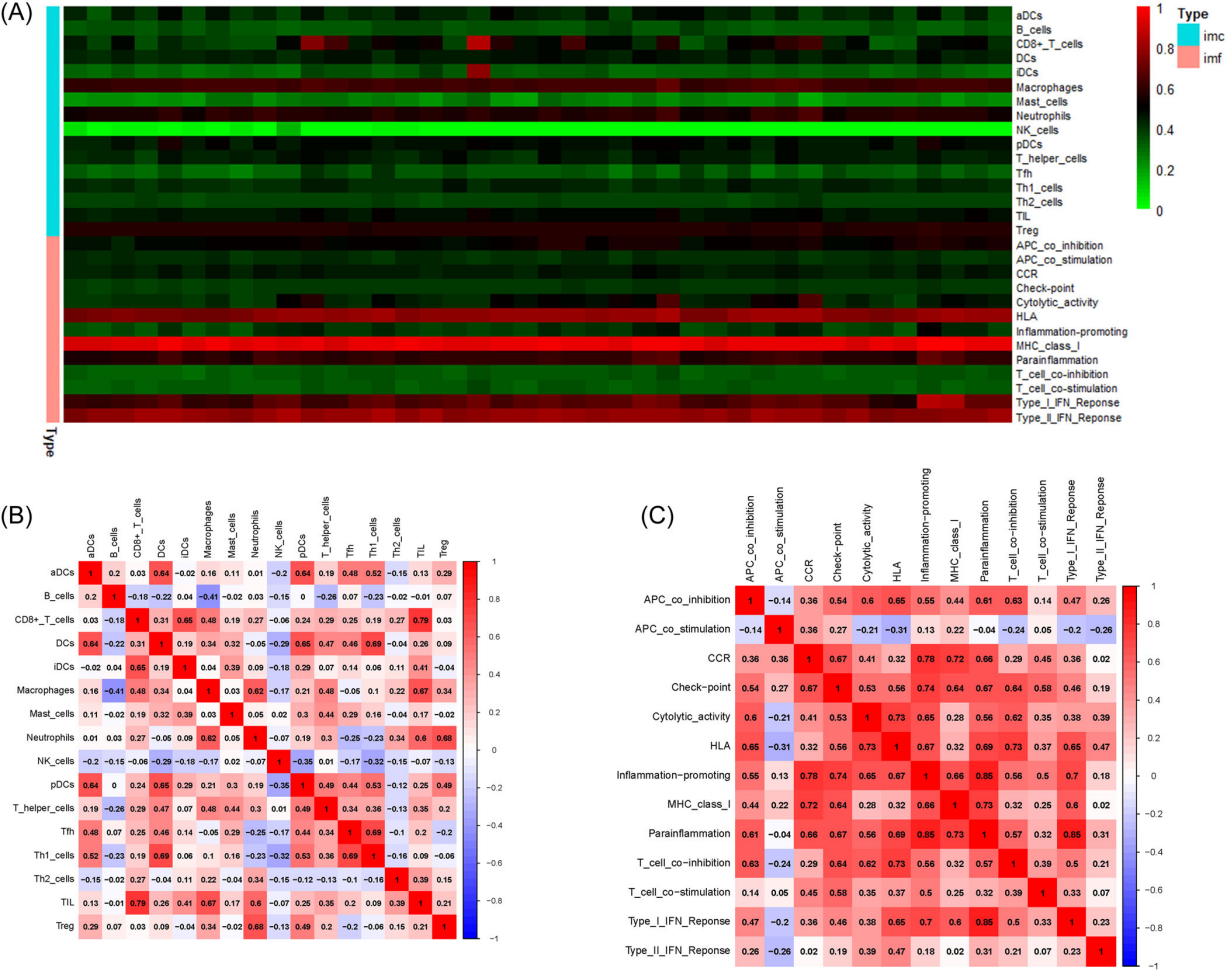
Heatmap and correlation analysis of immune cells and functions. (A) The heatmap of immune cells and functions. (B) Correlation analysis of immune cells. (C) Correlation analysis of immune functions.

More specifically, we used ssGSEA and GSVA to quantify the enrichment scores of various immune cell subpopulations, associated functions, or pathways and the links between risk score. Figure 7A shows the heatmap of immune cells and functions.

Figure 7B revealed correlation heatmap of the 15 types of immune cells. CD8+ T cell has a high positve correlation with TIL. DCs has a high positve correlation with Th1 cell macrophages has a high positve correlation with Til. Neutrophils has a high positve correlation with Tregs. Tfh has a high positve correlation with Th1 cell.

Figure 7C revealed correlation heatmap of the 13 types of immune funtions. CCR has a high positve correlation with inflammation‐promoting and MHC class‐I. Check‐point has a high positve correlation with inflammation‐promoting. Cytolytic‐activity has a high positve correlation with HLA. HLA has a high positve correlation with T cell coinhabitation. Inflammation‐promoting has a high positve correlation with parainflammation. Inflammation‐promoting has a high positve correlation with type I IFN response.

**Figure 8:**
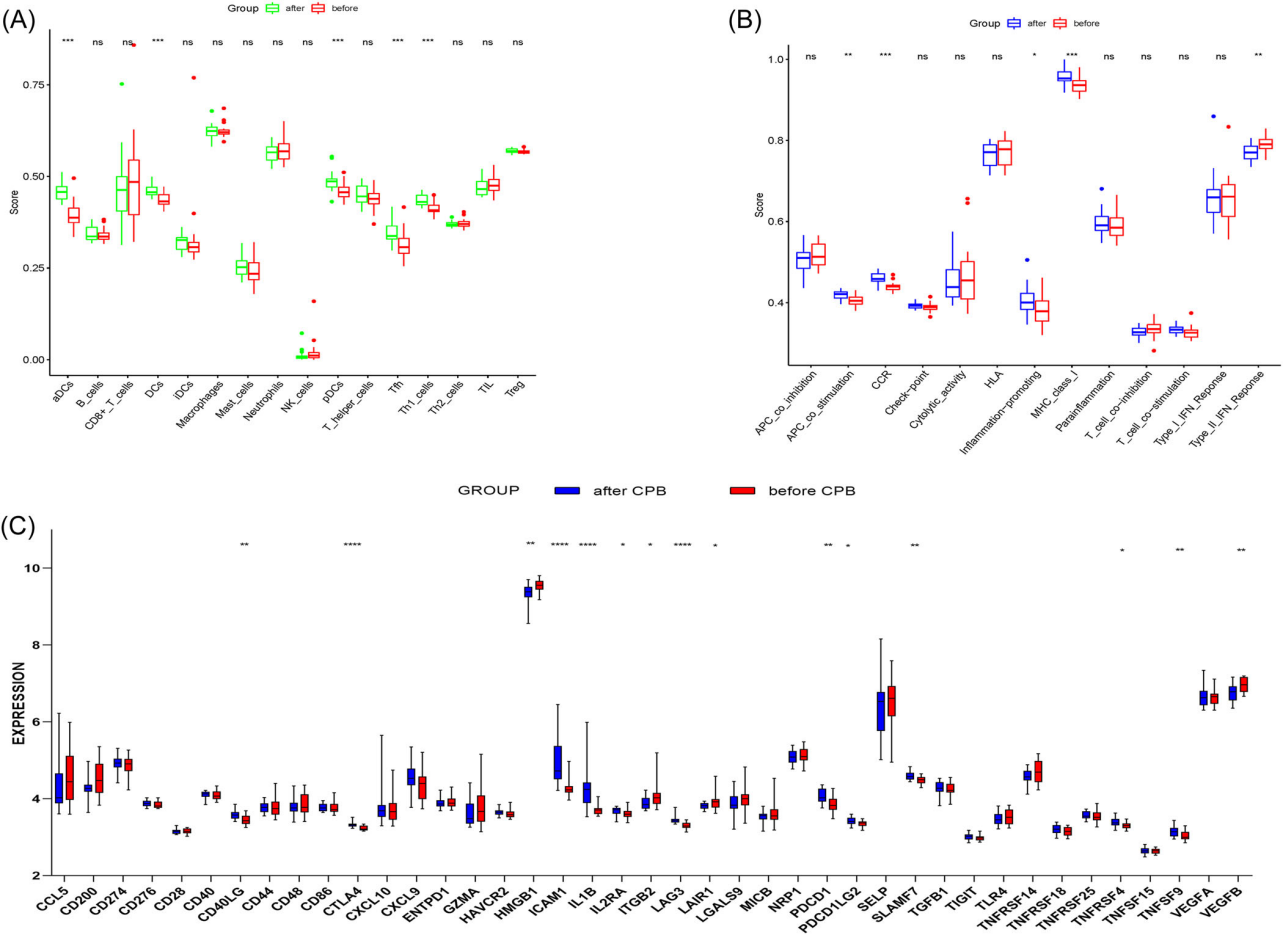
Analysis of different immune cells, immune function, and immune checkpoint in immune infiltration. (A) Boxplot of the proportion of immune cells. (B) Boxplot of the proportion of immune function. (C) Boxplot of the proportion of immune checkpoint (*p < .05, **p < .01, ***p < .001).

The boxplot of the immune cell infiltration (Figure 8A−C) difference showed that compared with the samples before CPB, immune cells such as aDCs (*p* < .001), DCs (*p* < .001), pDCs (*p* < .001), Tfh (*p* < .001), Th1 cells (*p* < .001), and immune functions such as APC‐CO‐stimulation (*p* < .01), CCR (*p* < .001), inflammation promoting (*p* < .05), MHC class I (*p* < .001), together with immune checkpoint CD40LG (*p* < .01), CTLA4 (*p* < .01), ICAM1 (*p* < .01), IL1B (*p* < .01), IL2A (*p* < .01), LAG3 (*p* < .05), PDCD1 (*p* < .01), PDCD1LG2 (*p* < .05), SLAMF7 (*p* < .01), TNFRSF4 (*p* < .05), and TNFSF9 (*p* < .01) increased while immune functions type II IFN‐response and immune checkpoint such as HMGB1, ITGB2, LAIR1, and VEGF decreased.

### 3.5 Enrichment analysis, medication prediction, correlation between CRGs and immune infiliatration

**Figure 9:**
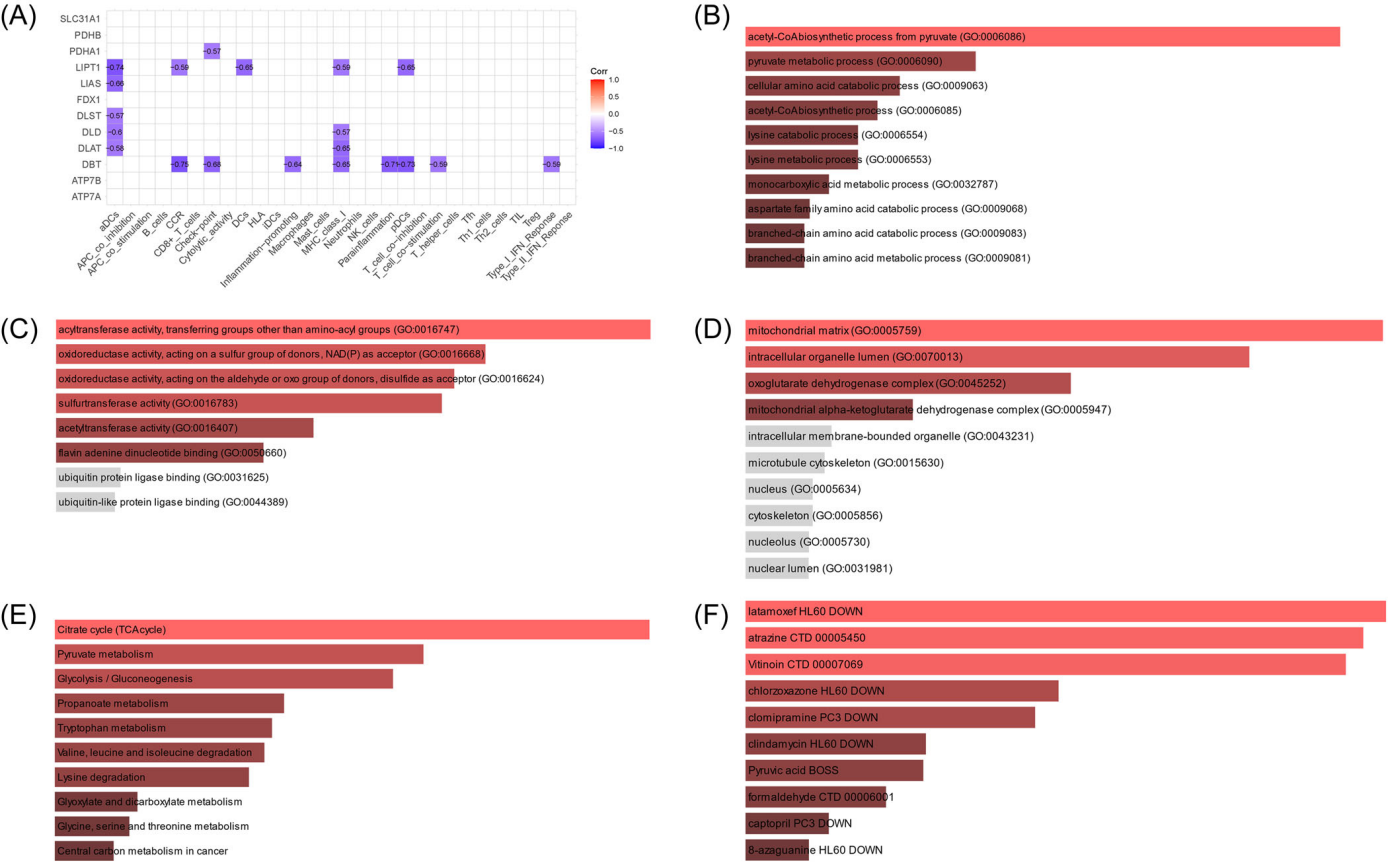
Cuproptosis and immune cell infiltration in the correlation analysis and Functional analysis and medication prediction. (A) LIPT1, LIAS, DLST, DLD DLAT, DBT, PDHA1 are negatively correlated with the corresponding immune cells and immune function. (B−D) The enriched item in the gene ontology analysis of DEGs. (A) BP: biological process, (B) CC: cellular component, (C) MF: molecular function. (E) The enriched item in the Kyoto encyclopedia of genes and genomes analysis. (F) The top 10 DsigDB prediction enrichment results. DEGs, differential genes.

LIPT, LIAS, DLST, DLD, DLAT, DBT, and PDHA1 are all negatively associated with immune cells and functions associated with cuproptosis and the details are as follows (Figure 9A).

LIPT1, LIAS, DLST, DLD, and DLAT are negatively correlated with cuprotosis‐related genes and immunological infiltration of aDCs and the DCs had an inverse relationship to LIPT1. In addition the pDCs have an inverse relationship to the DBT and DBT and T cell costimulation are in an inverse relationship. What's more, DBT also had a negative connection with proinflammatory factors and it is shown that CCR is inversely related to LIPT1, DBT. Next, there is an inverse relationship between MHC class I and LIPT1, DLD, DLAT, and DBT and between parainflammation and DBT. However, There is also an inverse relationship between CHECKPOINT and PDHA1 and between type I IFN response and DBT.

The enrichment analysis of CRGs related to immune cells and functions is similar to The enrichment analysis of CRGs identified (Figure 9B−E). Top 10 of predicted medications involved in copper‐related cell deaths are latamoxef (*p* < .05), atrazine (*p* < .05), vitinoin (*p* < .05), chlorzoxazone (*p* < .05), clomipramine (*p* < .05), clindamycin (*p* < .05), pyruvic acid (*p* < .05), formaldehyde (*p* < .05), captopril (*p* < .05), and 8‐azagyabure (*p* < .05) (Figure 9F).

## 4 DISCUSSION

In our study, we explore the likely function of immune infiltration and genes related with cuproptosis in heart tissue injury following CPB for the first time. Despite the substantial risk of postoperative inflammation and organ injury, CPB are becoming more and more popular in treating congenital heart defects.^19^, ^20^, ^21^, ^22^, ^23^, ^24^ Clinically unfavorable outcomes of cardiopulmonary bypass might range from minor symptoms to life‐threatening complications and heart failure and low cardiac output syndrome is the most severe consequence.^25^ Conventional myocardial protection during surgical procedures focuses on reducing the inflammatory response^26^ which is consistent with our enrichment analysis results. In addition to evaluating the significance of inflammation and the immune system, we perform an immune infiltration, the results of which reveal the significance of dendritic cells, Th1 cells, Apc CO inhibition, CCR‐inflammation promotion, MHC class I, and type II IFN responses. In the immune cell infiltration, previous research has revealed that after an acute myocardial infarction, DC are present in cardiac tissue and are related with significantly elevated HLA‐DR levels.^27^ In the meanwhile, T‐helper cell differentiation and maturity are promoted in patients with heart failure, but B cell toxic cells are diminished.^28^ This indicated that the combination of DCs and T cells may induce an immunological response that exacerbates cardiac injury. As to immune function infiltration, interferons with type II response are useful for treating heart failure in clinical trials. What's more, on the basis of the direct effects of IFN on cardiac fibroblasts, Hellkvist et al. discovered that IFN caused the synthesis of hyaluronic acid and stimulated the development of fusion cultures including cardiac fibroblasts obtained from rat hearts.^29^

In addition to immunological and inflammatory factors, prior research also indicates that myocardial copper concentrations rose during the aortic cross‐clamping phase of heart surgery^30^ and copper may be liberated from extracorporeally circulating blood during cardiopulmonary bypass.^31^ Cuprotosis is a novel programmed cell death characterized by aberrant copper ion concentrations and abnormal citric acid cycle.^6^ As to abnormal citric acid cycle, Aaron K. Olson's study demonstrated that triiodothyronine increases myocardial function and pyruvate entry into the citric acid cycle after reperfusion in a model of infant cardiopulmonary bypass.^32^, ^33^

Advancements in transition metal signalings have facilitated the translation of basic studies in copper chemistry and biology into therapeutic drugs and diagnostics by establishing novel interconnections. During congenital heart disease repair surgery, ferroptosis, the new metal signalings, is connected with mitochondrial dysfunction in cardiac tissue^34^ and in our study, cuproptosis, another metal signaling, shows a strong correlation with mitochondrial damage. The differential CRGs in our study such as LIPT1, LIAS, and DLD (related genes of key enzymes of lipoic acid pathway) and DLAT and PDHB (related genes of pyruvate dehydrogenase complex) can significantly alleviate the cytotoxicity mediated by copper ion carrier where LIAS is the key genes in cuproptosis procedure.^7^ Maintaining proper pyruvate metabolism,^35^ anaerobic glycolysis, and glycogen decomposition increasing^36^ and glycolysis and ketooxidation rates lifting^37^ may be the reasons how the mitochondria was injuried.

Next, the data set is utilized to analyze the genes implicated in cuproptosis through immune infiltration; DBT, DLAT, DLD, LIPT1, and PDHC are demonstrated to have a significant inverse relationship with their related immune cells. As to the indicated clinical medicine, the DSigDB database of pharmacological compounds forests latamoxef, atrazine, vitinoin, chlorzoxazone, and clomipramine as essential medications. Latamoxef are evidence to perform anti‐inflammatory functions.^38^ Chlorzoxazone has negative correlation with plasma TNF and IL‐6 levels in patients with congestive heart failure.^39^ Moreover, clomipramine‐treated infected mice exhibited less cardiac inflammation, tissue necrosis, and fibrosis than untreated infected animals.^40^

This research presents a novel route of myocardial protection during CPB, despite significant limitation due to the limited sample size of the data set contained (10 instances with TOF and ASD) and the difference between TOF and ASD may also affect the result. Meanwhile due to the lack of sequences on myocardial injury after CPB, it is hard to add the verifying set to eliminate the difference in population and gene characteristics. Although there is no confirmed clinical evidence to support the DsiDB‐predicted medication, interfering with cuprotosis‐related cell death may extend the lives of CPB‐experienced individuals. Subsequent research are needed to examine single‐cell sequencing data from a larger population to better comprehend this phenomena.

## 5 CONCLUSIONS

Our research demonstrates that immune infiltration and cuproptosis are the potential mechanisms by which cardiopulmonary bypass surgery may injure heart tissue, which might be a unique medicinal development target.

## AUTHOR CONTRIBUTIONS

**Song Puwei**: Software and writing—original draft (equal). **Ma Siyu**: Methodology (equal). **DeQin ZhuoGa**: Software (equal). **Wu Kede**: Data curation (lead). **Yang Zhaocong**: Writing—original draft (lead). **Nishant Patel**: Writing—original draft (lead). **Liu Xiaoxu**: Software (lead). **Mo Xuming**: Writing—review & editing (equal).

## CONFLICT OF INTEREST STATEMENT

The authors declare no conflict of interest.

## ETHICS STATEMENT

The studies were reviewed and approved by the Ethics Committee of the Children's Hospital of Nanjing Medical University (IACUC‐ 1908019).

## Data Availability

The data that support the findings of this study are available from the corresponding author, upon reasonable request.
